# Treatment of Modified Dahuang Fuzi Decoction on Cognitive Impairment Induced by Chronic Kidney Disease through Regulating AhR/NF-*κ*B/JNK Signal Pathway

**DOI:** 10.1155/2022/8489699

**Published:** 2022-04-14

**Authors:** Mingjia Gu, Pu Ying, Zhiwei Miao, Xiang Yu, Rui Bao, Jian Xiao, Leiping Gao, Juping Chen

**Affiliations:** ^1^Changshu Hospital Affiliated to Nanjing University of Chinese Medicine, Changshu, Jiangsu 215500, China; ^2^Zhangjiagang TCM Hospital Affiliated to Nanjing University of Chinese Medicine, Zhangjiagang, Jiangsu 215600, China; ^3^Nanjing University of Chinese Medicine, Nanjing 210046, Jiangsu, China; ^4^The College of Pharmacy of Jiangsu University, Zhenjiang 212000, Jiangsu, China

## Abstract

**Aim:**

An increasing widespread of chronic kidney disease (CKD) has been established lately around the globe. In addition to renal function loss, CKD can also cause cognitive impairment (CI). Modified Dahuang Fuzi Decoction (MDFD) is used as a traditional Chinese therapy for CKD. The effect of MDFD on cognitive impairment induced by chronic kidney disease (CKD-CI), and therapeutic mechanisms were investigated.

**Methods:**

The CKD animals' model was developed in the 5/6 nephrectomized mice. Sham operation and model groups received normal saline, while positive control and MDFD high/medium/low dose received Aricept (10 mg/kg/day) and different doses of MDFD (24, 16, and 8 g/kg/day), respectively. Cognitive function was detected with the Morris water maze test, while related factors were determined by ELISA. Histopathology and mechanism were studied using HE, western blot, and qRT-PCR.

**Results:**

In the CKD-CI mice model, escape latency decreased significantly, whereas time of crossing platform and time spent within the platform quadrant increased substantially (*P* < 0.05) after MDFD treatment. Moreover, renal function and brain injury in CKD-CI improved dose-dependently, while the effect of MDFD-L was worse. Proteins such as aryl hydrocarbon receptor, nuclear factor-kappa B and c-Jun-N-terminal kinase, and mRNA in the kidney and brain of all the treatment groups decreased substantially (*P* < 0.05). Expression of tropomyosin receptor kinase B and brain-derived neurotrophic factor at protein and mRNA levels in the brain were significantly enhanced (*P* < 0.05).

**Conclusion:**

MDFD presumably activated the BDNF/TrkB pathway by inhibiting the AhR/NF-*κ*B/JNK signaling pathway to treat CKD-CI.

## 1. Introduction

Malformation of renal structure or function lasting over 3 months culminate in chronic kidney disease (CKD), which is a disease process in which the renal function declines slowly and finally goes to failure. With the advent of an aging population, the incidence rate of CKD is concomitantly increasing year by year. The disease is now a grave menace to public health globally. Compared with nonkidney disease patients, there is a strong likelihood that CKD patients will suffer from cognitive impairment (CI). Affirmative to this, CI incidence in CKD patients who underwent maintenance hemodialysis was determined to be 85% [1]. Another study also showed that nearly 75% of CKD patients who received peritoneal dialysis had CI [2]. It is reported that CI may develop into dementia when it is not effectively treated and has the potential to greatly increase the rates of hospitalization, disability, and mortality among CKD patients [3]. At present, treatment of the disease mainly focuses on the diagnosis and treatment of “dementia” in the department of neurology, with cholinesterase inhibitors as the principal therapy, albeit insufficient research on its safety and long-term prognosis [4]. Currently, CI complications in CKD patients are difficult to treat in the clinics.

The exact mechanism of CI occurrence in CKD patients has not been fully elucidated. Some complications of CKD, namely, renal anemia, secondary hyperparathyroidism, hemodynamic changes, and dialysis imbalance, are considered to be closely related to the disease [5]. As an important feature of CKD, toxin accumulation has always been considered as an important cause of CI. With more and more attention been paid to the cognitive function of CKD patients in recent years, some scholars have suggested indoxyl sulfate (IS) to be an important toxin which is causing CI in CKD patients, and hence, its potential therapeutic target should be studied [6]. As a type of nephro-vascular toxin, IS comes from tryptophan decomposition in food with 90% of IS binding to protein. Compared with normal people, the concentration of IS in CKD patients is significantly higher [7]. Because IS can increase the production of inflammatory reaction and oxidation products [8], it is likely that IS is the initiating factor of CI in CKD patients [9]. Negative correlation between serum level of IS and cognitive function of CKD patients was observed by Yeh [10]. Lin found that cognitive function (including long-term memory, mental control, language ability, and visuospatial structure) of CKD patients who underwent maintenance hemodialysis correlated negatively with serum-free IS level [11].

From the perspective of traditional Chinese medicine, chronic kidney disease is a long course condition and often has damaged viscera function which results in abnormal operation of Qi and blood, thereby culminating in accumulation of pathological products such as phlegm dampness, blood stasis, turbid toxin, destruction of brain, invasion of nerves, and cognitive dysfunction. Modified Dahuang Fuzi Decoction (MDFD) is a long-term prescription of Professor Zou Yunxiang (Nanjing University of traditional Chinese Medicine) for CKD treatment. This prescription evolved from the famous Dahuang Fuzi Decoction (comprising *Radix Aconiti Lateralis Praeparata*, *Radix et Rhizoma Rhei*, and *Asarum*) which has been recorded in the Synopsis of the Golden Chamber written by Zhang Zhongjing, a doctor of the Han Dynasty. Its main function is to excrete toxins in patients with CKD and improve renal function and a series of complications.

Therefore, the effect of MDFD on alleviating CI via improvement of renal injury was investigated. The model of cognitive dysfunction induced by kidney injury was established in mice, and they were treated with MDFD via oral administration. Verification of curative effect of MDFD on CKD-CI was carried out. The cognitive function, renal function index, and hippocampal inflammation index of mice after treatment were investigated. Additionally, this work sought to confirm the correlation between the therapeutic effect and the metabolism of IS, as well as explore mechanistic action of MDFD.

## 2. Materials and Methods

### 2.1. Drugs and Reagents

Tianjiang Pharmacology Co. Ltd (Jiangyin, China) provided *Radix Aconiti Lateralis Praeparata*, *Concha Ostreae*, *Radix et Rhizoma Rhei*, *Herba Taraxaci*, *Flos Sophorae Immaturus*, and *Serissa serissoides Druce*, whereas the China Institute for the Control of Pharmaceutical and Biological Products (Beijing, China) supplied all other related analytical reference (98% purity). Kidney injury factor-1 (KIM-1), serum creatinine (SCr), blood urea nitrogen (BUN), *β*2-microglobulin (*β*2-MG), malondialdehyde (MDA), superoxide dismutase (SOD), tumor necrosis factor alpha (TNF-*α*), and interleukin-1 *β* (IL-1*β*) ELISA kits were bought from the Nanjing JianCheng Bioengineering Institute (Nanjing, China). Abcam corporation (Cambridge, USA) supplied antibodies for aryl hydrocarbon receptor (AhR), anti-phospho-c-Jun-N-terminal kinase (JNK) cell signaling, nuclear factor-*κ*B (NF-*κ*B), antitropomyosin receptor kinase B (TrkB), and anti-brain-derived neurotrophic factor (BDNF). All other reagents and substances were kindly provided by Sigma Chem. Comp., (Sigma, Milan, Italy) unless otherwise stated.

### 2.2. Experimental Subjects

#### 2.2.1. Animal's Source

Ninety SPF grade male C57 mice (25 ± 2 g) were purchased from the Yangzhou University Comparative Medicine Center. Before the experiment, all the mice were acclimatized to controlled condition, viz., temperature (22 ± 3°C), humidity (50 ± 10%), and a cycle of 12 h light and 12 h darkness for 1 week.

#### 2.2.2. Ethical Statements

The Ethics Committee at Jiangsu University approved the animal experiments while the principle for the Use of Laboratory Animals was based on the European Community Guidelines.

### 2.3. Preparation of MDFD

The composition of MDFD included *Radix Aconiti Lateralis Praeparata* (10 g), *Radix et Rhizoma Rhei* (20 g), *Concha Ostreae* (40 g), *Flos Sophorae Immaturus* (30 g), *Herba Taraxaci* (30 g), and *Serissa serissoides Druce* (30 g). All the herbs were put into a round bottom flask and water-soaked for 30 min. Next, they were decocted and extracted twice. The original solution was collected by filtering while hot with the collection of the filtrate being done twice prior to concentration to 1 g/mL crude drug volume. The pH of the liquid was then adjusted to 7.0, and the MDFD was subsequently stored at 4°C (for further experimental use) after removal of bacteria via filtration.

### 2.4. LC-MS Analysis on Major Compounds of MDFD

An LC-MS was applied to analyse the main chemical components of MDFD. The conditions for the LC-MS analysis had shim-pack XR-ODS C18 (1.6 *μ*m, 2.0 × 75 mm) as chromatographic column, acetonitrile (A): water (B), at the following eluting gradient: 0–120 min, (A) 2%-(B) 98% as mobile phase, 30°C as column temperature, 1.0 mL/min as flow rate, 2 *μ*L as injection volume, and 280 nm as the detection wavelength. Other conditions considered were ESI positive and negative ion mode, 15 kV capillary voltage, 3 L/min atomizer flow, 10 L/min heat gas flow, 350°C interface temperature, 250°C DL temperature, 400°C heating block temperature, 10 L/min drying gas flow, and M/Z 200∼1000 scanning range. According to the established chromatographic and mass spectrometric conditions, the prepared MDFD and each reference solution (100 *μ*g/mL) were injected to obtain the LC-MS total ion flow diagram.

### 2.5. Establishment and Grouping of the CI Mice Model

On the account of random number table, randomization of the mice into sham operation (S), model (M), positive control (P), MDFD high-dose (MDFD-H), MDFD medium-dose (MDFD-M), and MDFD low-dose (MDFD-L) groups was performed with 15 mice in each group. Based on the previous method [12], a mouse model of CKD was established by 5/6 nephrectomized. The mice in each group were fasted for 8 h before operation, and the mice were weighed and recorded. Anesthesis of the mice in the groups M, P, MDFD high, medium, and low dose was carried out by intraperitoneally injecting them with chloral hydrate (4%, 10 g/mL) and before placing them on the operating table. An oblique incision was made at 1 cm below the left rib with the incision forming an angle of 45° inward with the longitudinal axis of the mouse body to expose the right kidney. After separating the fat around the kidney and the outer capsule of the kidney, 1/3 of the renal upper and lower pole tissues were removed. Compression of the hemostasis for 3 min, prior to resetting and suturing of the kidney was performed with gelatin sponge. Next, 80000U penicillin was injected intraperitoneally to prevent infection. After the above-described operation, a complete resetting of the left kidney was observed after one week. Penicillin (80000U) was intraperitoneally injected into the mice and were given water and observed carefully. In the sham operation group, only the left and right kidneys were exposed and the perirenal fat and capsule were separated and sutured. After the operation, the mice were placed on a 37°C heat preservation blanket and were reared normally after resuscitation.

### 2.6. Morris Water Maze (MWM) Experiment

The mice with CKD were fed for another 2 weeks. The MWM test was performed to determine the cognitive function of mice in each group and ascertain whether the cognitive impairment model was successfully established. Assessment of mice CI in the MWM was based on previous work [13]. The instrument for the experiment comprised a 150-cm diameter circular pool with a water depth of 40 cm, while a temperature maintained at 25 ± 2°C. In addition, a 6-cm dimensioned escape platform was placed in a quadrant of the circular pool. Video behavioral analysis system (SMART 3.0, Spanish Panlab Company) was used to record the trajectory data of mice for the extraction and analysis of various indexes.

The experiment lasted about 6 days and consisted of 5-day place navigation (3-day training and 2-day formal testing) and 1-day navigational memory tests. The place navigation test mainly reflected the spatial learning and executive function of mice. From the first day, put the mouse facing the pool wall into the pool from four quadrants. Starting position was the quadrant that was opposite to the escape platform quadrant. Later, the time at which the mice boarded the escape platform was recorded; thus, this was considered as the escape latency (EL). The spatial learning ability of the mice was regarded as worse when the EL was larger. Training of each mouse was done four times within a day for three days with 20 min interval between each training. During the training phase, the platform was exposed to 0.5 cm above the water surface. When a mouse was unable to locate the escape platform within 120 s, the experimenter would guide it to the platform and let the mouse stay on the platform for 15, and the EL value was maximum (120 s) at this time. On the 4^th^ and 5^th^ day of formal test, the platform was hidden 0.5 cm under the water, while milk was poured into the water surface to make the platform invisible. Each mouse was tested once a day to obtain two-day average EL value. Next, a day's navigation memory test was employed to test the position memory ability of mice. 40 cm deep water was put into the pool, and the escape platform was withdrawn from the pool. Afterwards, the mice were put into the water towards the pool wall, and recording of the time spent in platform quadrant (TSPQ) was done within 120 s. Times for crossing platform (TCP) were recorded as well amidst each mouse being tested only once.

### 2.7. Administration Method

**Figure 1:**
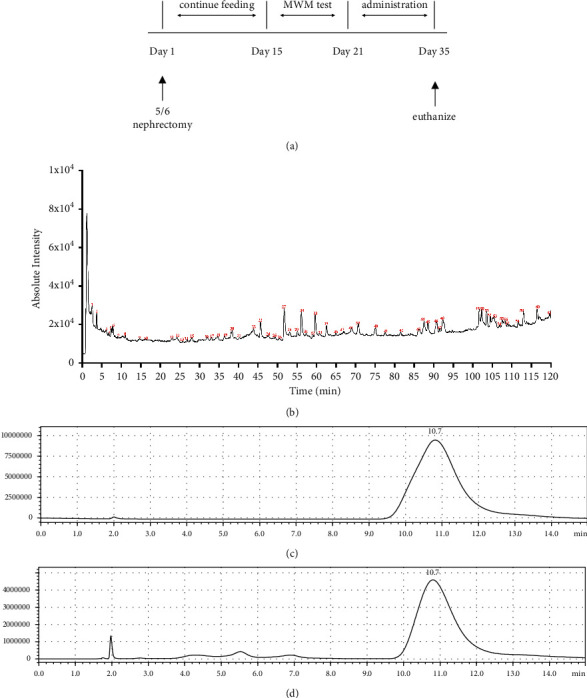
(a) Animals experimental process. (b) Total ion flow diagram of high-performance liquid chromatography-mass spectrometry (LC-MS). (c) HPLC chromatogram of indoxyl sulfate standard. (d) Mixture of indoxyl sulfate and blank serum of mice.

After the combined model of CKD and CI was successfully established, groups (MDFD-H, MDFD-M, and MDFD-L) were given MDFD at respective doses of 24, 16, and 8 g/kg by gavage per day. Groups S and M received the same amount of normal saline, while group P was given Aricept (dissolved in the same amount of normal saline, 10 mg/kg/day) by gavage. The cognitive function was recorded after two weeks of treatment using the MWM experiment. Blood was taken from eyeballs of the mice into heparin-containing tubes after being euthanized, while the kidney and brain tissues were simultaneously collected. The whole experimental process is simply shown in Figure 1(a).

### 2.8. HPLC Method of IS in Serum

High performance liquid chromatography fluorescence method (HPLC-FLU) was used for the concentration determination of IS in serum. The standard reference substance of potassium indoxyl sulfate (23.71 mg) (equivalent to 20 mg of indoxyl sulfate) was accurately weighed, then put into a 25-mL volumetric flask, dissolved with methanol, and diluted to the scale to obtain 800 *μ*g/mL stock solution. The solution was stored in a refrigerator at 4°C for standby. Aliquot (100 *μ*L) of blank serum of mice and appropriate amount of standard stock solution were taken to prepare the sample with the serum concentration of IS of 80, 40, 20, 10, 5, 0.5, and 0.1 *μ*g/mL. Next, 300 *μ*L 0.2% trifluoroacetic acid acetonitrile (10 : 90) solution was added into the sample to precipitate the protein, vortexed for 30 sec, and centrifuged at 4°C at 12000 rpm/min for 8 min. Afterwards, 20 *μ*L of supernatant was collected for injection analysis, while the chromatogram was recorded.

The chromatographic conditions were as follows: the mobile phase was sodium dihydrogen phosphate buffer (containing 0.1% trifluoroacetic acid, pH2.5) acetonitrile (92 : 8, v/v), the flow rate was 1.0 mL/min, the column temperature was kept at 30°C, the sample temperature was 6°C, the excitation wavelength was 280 nm, and the emission wavelength was 390 nm. The concentration of IS served as the abscissa, and the peak area of IS was the ordinate. Finally, the linear regression equation obtained was the standard curve of IS. The peak area was substituted into the standard curve to calculate the serum concentration of IS.

### 2.9. Detection of IS and Related Factors in the Blood, Kidney, and Brain of Mice

Next, treatment of samples of blood was carried out as stated in the method in “2.8 HPLC method of IS in serum”. Mice kidney and brain tissue samples were mixed with normal saline at the ratio of 1 : 5 (w/v), prior to obtaining the suspension via homogenizer. Centrifugation of all the samples, namely, serum, kidney, and brain was performed (10 min) at 3000 rpm to promptly obtain the supernatant for storage at −20°C pending future experiment. The serum levels of BUN, SCr, KIM-1, *β*2-MG, SOD, MDA, TNF-*α*, and IL-1*β*, coupled with KIM-1, *β*2-MG, MDA, SOD, TNF-*α*, and IL-1*β* levels in kidney, as well as concentrations of MDA, SOD, TNF-*α*, and IL-1*β* in the brain of mice were determined appropriately in compliance with standard operating procedures of ELISA kits. The concentration of IS in serum of mouse was detected via HPLC-FLU method as mentioned above.

### 2.10. Histopathological Examination

After the mice were sacrificed, the hippocampus and kidney were separated and stored in 10% formalin solution. After dehydration and embedding, the tissues were cut into 4-*μ*m thick paraffin sections for staining with hematoxylin & eosin (HE). Afterwards, the prepared samples were observed under a microscope (Nikon, Japan) for any pathological changes.

### 2.11. Protein Content in Mice

The WB test was performed to detect the protein content of AhR, NF-*κ*B, JNK, BDNF, and TrkB proteins in brain and NF-*κ*B, AhR, and JNK proteins in kidney. Then, extraction of protein from protein lysate was done and quantified with an ultraviolet spectrophotometer. The volume of each pore sample was 50 *μ*g, while it was electrophoresised in 5% 12 alkyl sulfate poly-acryl-amide gel electrophoresis (SDS-PAGE) before immediate transfer to the PVDF membrane. After the transfer, PBS containing 5% skimmed milk powder was applied as blocking agent at ambient temperature for 2 h. Corresponding first antibody (1 : 500) was added and incubated for 5 min at 4°C. Next, addition and incubation of the second antibody (dilution ratio 1 : 500) was done for 1 h at ambient temperature. Consequently, ECL chromogenic fluid was added to the washing film for 5 min. The gray value of the protein strip with the gel image processing system was analyzed. The ratio of gray value of target protein to reference internal protein was expressed as the relative expression of protein. Moreover, Methods and Results of Immunohistochemistry and Immunofluorescence are provided in the supplementary file.

### 2.12. Quantitative Reverse Transcription Polymerase Chain Reaction (qRT-PCR)

Trizol reagent (Invitrogen, Carlsbad, CA, USA) was employed to extract total RNA from tissues of kidney and brain. Total RNA 10 pg–100 ng was added into a 0.2-mL PCR tube. The reverse transcription of cDNA was carried out based on the specifications of the kit's manufacturer (Invitrogen, Carlsbad, CA, USA). Then, RT-PCR was carried out with an Applied Biosystem using SYBR Green (Takara, Japan). The 96-well PCR plate was covered with a sealing film (special for fluorescence quantitative analysis) and then put into fluorescence quantitative PCR instrument after instant centrifugation. The conditions for the reaction were set for 2 min at 95°C (after predenaturation) and 20 s at 94°C to 20 s at 60°C and 20 s at 68°C (40 cycles), where the data were saved after reaction. Standard curve was drawn after confirming melting and amplification curves of qRT-PCR.

### 2.13. Statistical Methods

Mean ± SD was used to express the data, which were analyzed with SPSS 18.0 software. Comparison of two groups was done with the *t*-test, while multiple group comparison was done with ANOVA (one-way). Acceptable significant difference was when the *P*-value was <0.05.

## 3. Results

### 3.1. Major Compounds of MDFD

The LC-MS total ion flow diagram of MDFD is shown in Figure 1(b). The main chemical components of MDFD can be determined by comparing the ion peaks of MDFD with the reference solution or existing literature. We basically identified 61 main chemical components in MDFD.

### 3.2. MWM Experiment

**Figure 2:**
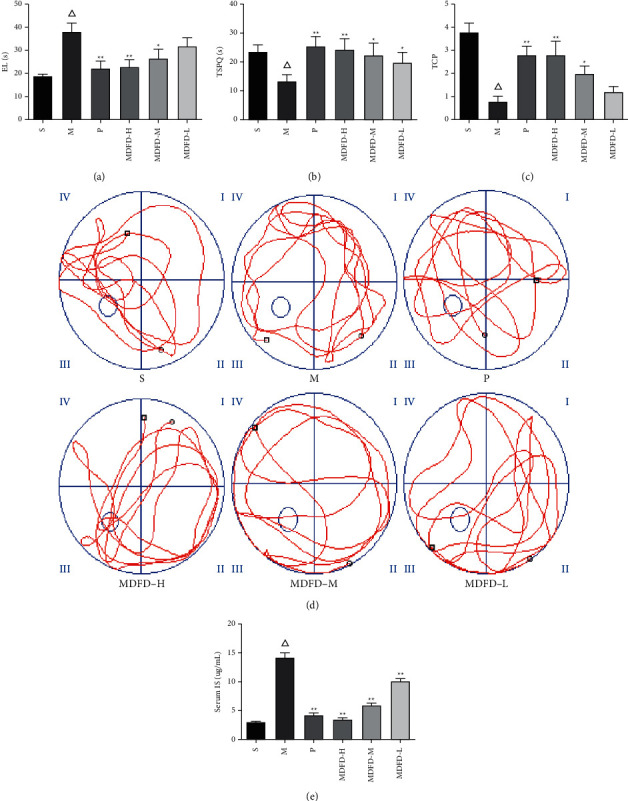
(a) EL of mice in all groups. (b) TSPQ of mice in all groups. (c) TCP of mice in all groups. (d) Trajectory of the times crossing the platform in all groups. (e) Serum IS concentration of mice in all groups (Δ*p* < 0.01, comparable to the S group; ^*∗∗*^*p* < 0.01, comparable to the M group; ^*∗*^*p* < 0.05, comparable to the M group; EL, escape latency; TSPQ, time spent in platform quadrant; TCP, times of crossing platform; S, sham operation group; M, model group; P, positive control group; MDFD-H, modified Dahuang Fuzi Decoction high-dose group; MDFD-M, modified Dahuang Fuzi Decoction medium-dose group; MDFD-L, modified Dahuang Fuzi Decoction low-dose group).

Figures 2(a)–2(c) display that EL of group M increased remarkably from 18.88 ± 1.62 s to 38.00 ± 8.46 s, while TCP (vs.) and TSPQ (vs.) of group M decreased significantly compared to group S, indicating that the CI model was successfully reproduced in this experiment (*P* < 0.05), and the trajectory of the number of times animals crossed the platform in the MWM test is shown in Figure 2(d). Comparable to group M, the EL of P (22.20 ± 7.19 s), MDFD-H (22.80 ± 6.80 s), and MDFD-M (26.6 ± 8.79 s) groups substantially decreased (*P* < 0.05), but TCP (2.80 ± 0.84, 2.80 ± 1.30, and 2.00 ± 0.71) and TSPQ (25.28 ± 7.82 s, 24.40 ± 8.32 s, and 22.40 ± 9.24 s) increased significantly (*P* < 0.05), albeit insignificant difference in the EL of MDFD-L and M groups (*P* > 0.05). The data showed that MDFD could improve the CI of CKD mice.

### 3.3. Detection of Serum IS and Related Factors in the Blood, Kidney, and Brain of Mice

The IS standard, mixture of IS, and blank serum of mice were analyzed under the chromatographic conditions in this study, wherein the chromatograms were obtained, respectively (Figures 1(c) & 1(d)). Other endogenous substances in serum did not interfere with the determination of IS. The peak shape of IS was good with a retention time of about 10.7 min. The standard curve equation of IS was *Y* = 23194X-7 × 10^7^ (*R*^2^ = 0.998). The results showed that the concentration of IS in serum had a good linear relationship in the range of 0.1∼80 *μ*g/mL.

As shown in Figure 2(e), and comparable to S, the serum concentration of IS in mice of group M markedly raised (3.04 ± 10.24 *μ*g/mL vs. 14.28 ± 1.61 *μ*g/mL, *P* < 0.05). Noteworthy, in comparison with group M, serum IS concentrations of P (4.26 ± 0.73 *μ*g/mL), MDFD-H (3.49 ± 0.48 *μ*g/mL), MDFD-M (5.94 ± 0.86 *μ*g/mL), and MDFD-L (10.17 ± 0.86 *μ*g/mL) groups markedly decreased (*P* < 0.05), notwithstanding, high-dose MDFD showed greater effect on the reduction of serum IS concentration.

**Figure 3:**
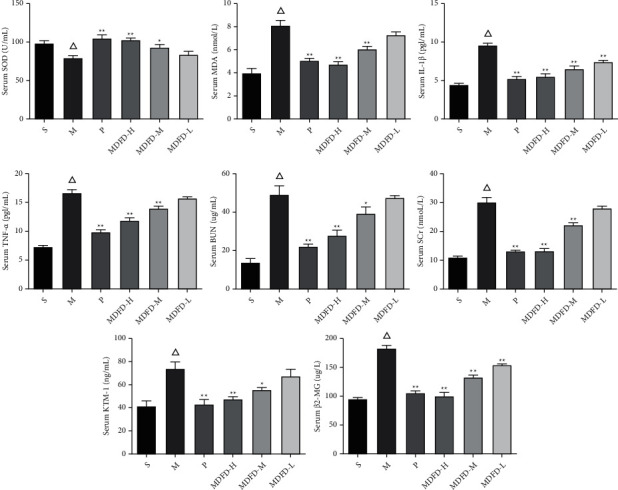
Related factors in mice serum of all groups (Δ*p* < 0.01, comparable to the S group; ^*∗∗*^*p* < 0.01, comparable to the M group; ^*∗*^*p* < 0.05, comparable to the M group; S, sham operation group; M, model group; P, positive control group; MDFD-H, modified Dahuang Fuzi Decoction high-dose group; MDFD-M, modified Dahuang Fuzi Decoction medium-dose group; MDFD-L, modified Dahuang Fuzi Decoction low-dose group; BUN, blood urea nitrogen; SCr, serum creatinine; KIM-1, kidney injury factor-1; *β*2-MG, *β*2-microglobulin; MDA, malondialdehyde; SOD, superoxide dismutase; IL-1*β*, interleukin-1*β*; TNF-*α*, tumor necrosis factor-*α*).

**Figure 4:**
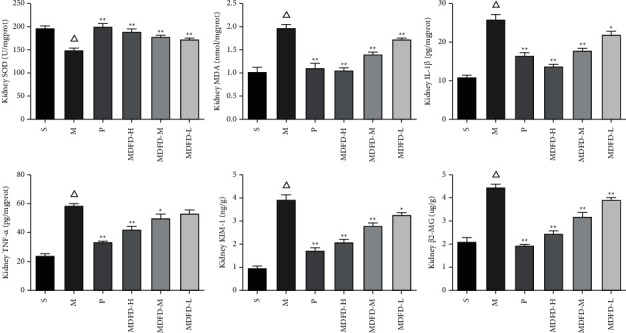
Related factors in mice kidney of all groups (Δ*p* < 0.01, comparable to the S group; ^*∗∗*^*p* < 0.01, comparable to the M group; ^*∗*^*p* < 0.05, comparable to the M group; S, sham operation group; M, model group; P, positive control group; MDFD-H, modified Dahuang Fuzi Decoction high-dose group; MDFD-M, modified Dahuang Fuzi Decoction medium-dose group; MDFD-L, modified Dahuang Fuzi Decoction low-dose group; KIM-1, kidney injury factor-1; *β*2-MG, *β*2-microglobulin; MDA, malondialdehyde; SOD, superoxide dismutase; IL-1*β*, interleukin-1*β*; TNF-*α*, tumor necrosis factor-*α*).

**Figure 5:**
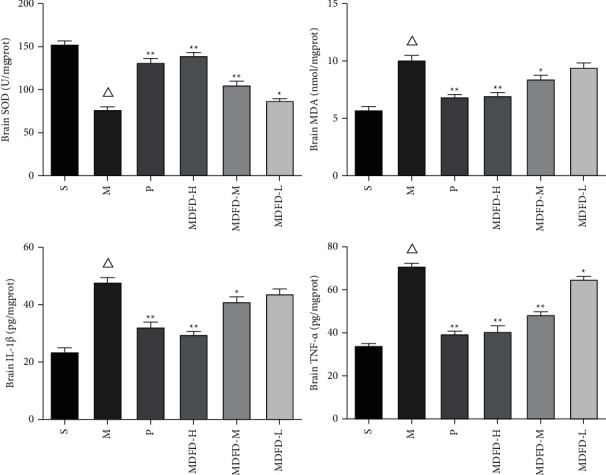
Related factors in mice brain of all groups (Δ*p* < 0.01, comparable to the S group; ^*∗∗*^*p* < 0.01, comparable to the M group; ^*∗*^*p* < 0.05, comparable to the M group; S, sham operation group; M, model group; P, positive control group; MDFD-H, modified Dahuang Fuzi Decoction high-dose group; MDFD-M, modified Dahuang Fuzi Decoction medium-dose group; MDFD-L, modified Dahuang Fuzi Decoction low-dose group; MDA, malondialdehyde; SOD, superoxide dismutase; IL-1*β*, interleukin-1*β*; TNF-*α*, tumor necrosis factor-*α*).

As described in Figures 3–5, a substantial reduction in SOD levels was found in serum, kidney, and brain of mice in the M group in comparison with S (*P* < 0.05). However, serum amounts of BUN, SCr, KIM-1, *β*2-MG, MDA, IL-1*β*, and TNF-*α* of group M were notably increased (*P* < 0.05) comparable to S. Similarly, the corresponding amounts of renal function related to physical and chemical indexes in kidney and brain of group M were also higher comparable to S (*P* < 0.05). Importantly, the activity of SOD was significantly increased, while MDA, IL-1*β*, and TNF-*α* concentrations were reduced markedly in the serum, kidney, and brain of groups (P, MDFD-H, and MDFD-M, *P* < 0.05) compared to group M. Besides, serum amounts of BUN, SCr, KIM-1, *β*2-MG, kidney and brain concentrations of KIM-1, and *β*2-MG in groups (P, MDFD-H, and MDFD-M) substantially reduced (*P* < 0.05) comparable to M group. The results stated clearly that different doses of MDFD could improve the renal function and brain injury in CKD mice with CI amidst the effect of low-dose MDFD being worst.

### 3.4. HE Staining

**Figure 6:**
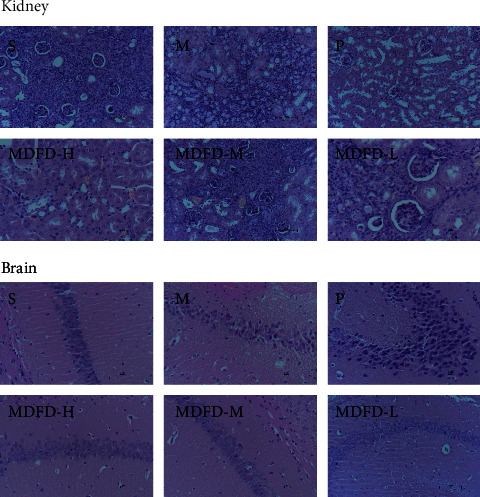
Pathological analysis of the mice kidney and brain in each group (S, sham operation group; M, model group; P, positive control group; MDFD-H, modified Dahuang Fuzi Decoction high-dose group; MDFD-M, modified Dahuang Fuzi Decoction medium-dose group; MDFD-L, modified Dahuang Fuzi Decoction low-dose group).

As exhibited in Figure 6, in the model group, inflammatory cell infiltration was obvious in renal interstitium with mesangial cells and stroma being moderately proliferated, while renal tubules were disordered. Compared with the model group, the inflammatory cell infiltration and renal interstitial hyperplasia decreased in the MDFD-H and MDFD-M groups. In MDFD-L group, mild glomerular edema and vacuoles of epithelia cells of renal tubular were observed. In addition, the results of pathological morphology of brain tissue (see Figure 6) in group S showed that the cell boundary of hippocampus tissue was clear and orderly. The neuronal numbers in hippocampus of mice in group M were less than that those in the sham operation, albeit fuzzy cell structure coupled with unclear boundary. After treatment with MDFD high-, medium-, and low-treated groups, the pathological morphology of mice brain tissue improved significantly in varying degrees. The neurons were arranged orderly, the cell structure was clear, and the nuclear membrane could be distinguished, while the nucleolus and cytoplasm were visible.

### 3.5. Related Protein Expression Levels in the Brain and Kidney

**Figure 7:**
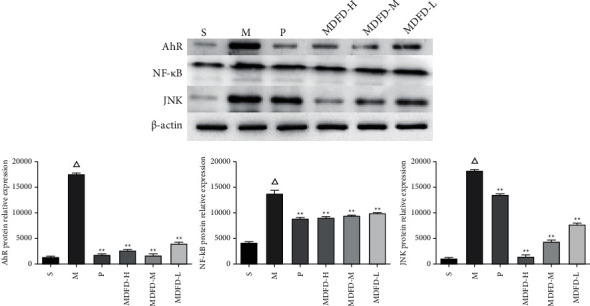
Western blot results of related protein expression levels in the mice kidney of all groups (Δ*p* < 0.01, comparable to the S group; ^*∗∗*^*p* < 0.01, comparable to the M group; ^*∗*^*p* < 0.05, comparable to the M group; S, sham operation group; M, model group; P, positive control group; MDFD-H, modified Dahuang Fuzi Decoction high-dose group; MDFD-M, modified Dahuang Fuzi Decoction medium-dose group; MDFD-L, modified Dahuang Fuzi Decoction low-dose group; AhR, aryl hydrocarbon receptor; NF-*κ*B, nuclear factor-*κ*B; JNK, c-Jun N-terminal kinase).

**Figure 8:**
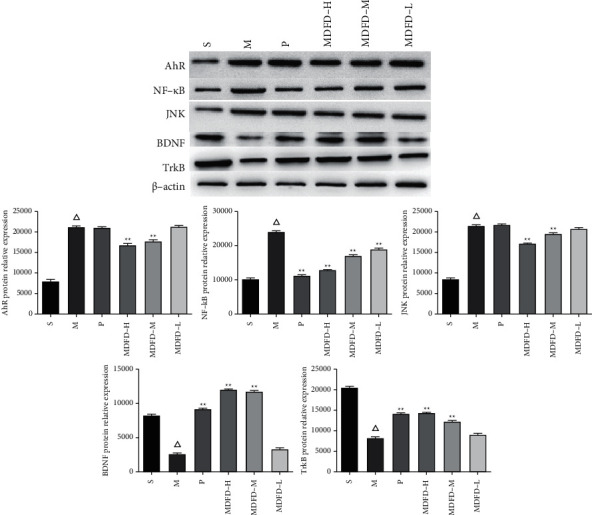
Western blot results of related protein expression levels in mice brain of all groups (Δ*p* < 0.01, comparable to the S group; ^*∗∗*^*p* < 0.01, comparable to the M group; ^*∗*^*p* < 0.05, comparable to the M group; S, sham operation group; M, model group; P, positive control group; MDFD-H, modified Dahuang Fuzi Decoction high-dose group; MDFD-M, modified Dahuang Fuzi Decoction medium-dose group; MDFD-L, modified Dahuang Fuzi Decoction low-dose group; AhR, aryl hydrocarbon receptor; BDNF, brain-derived neurotrophic factor; TrkB, tropomyosin receptor kinase B; NF-*κ*B, nuclear factor-*κ*B; JNK, c-Jun N-terminal kinase).

AhR, NF-*κ*B, and JNK expression (at protein level) in kidney of mice are shown in Figure 7. Compared with group S, the AhR, NF-*κ*B, and JNK protein content in CKD mice was increased. After the intervention of prednisone and different doses of MDFD, expression of AhR, NF-*κ*B, and JNK proteins in CKD mice decreased. As shown in Figure 8, compared with group S, AhR, NF-*κ*B, and JNK protein contents in the brain of group M increased. Also, AhR, NF-*κ*B, and JNK at protein level in the brain of the groups (P, MDFD-H, and MDFD-M) reduced in comparison with group M. In contrast, the BDNF and TrkB expression (at protein level) in group M decreased compared with group S, but it increased in groups (P, MDFD-H, and MDFD-M) compared to group M.

### 3.6. Expression Levels of Brain- and Kidney-Related mRNA

**Figure 9:**
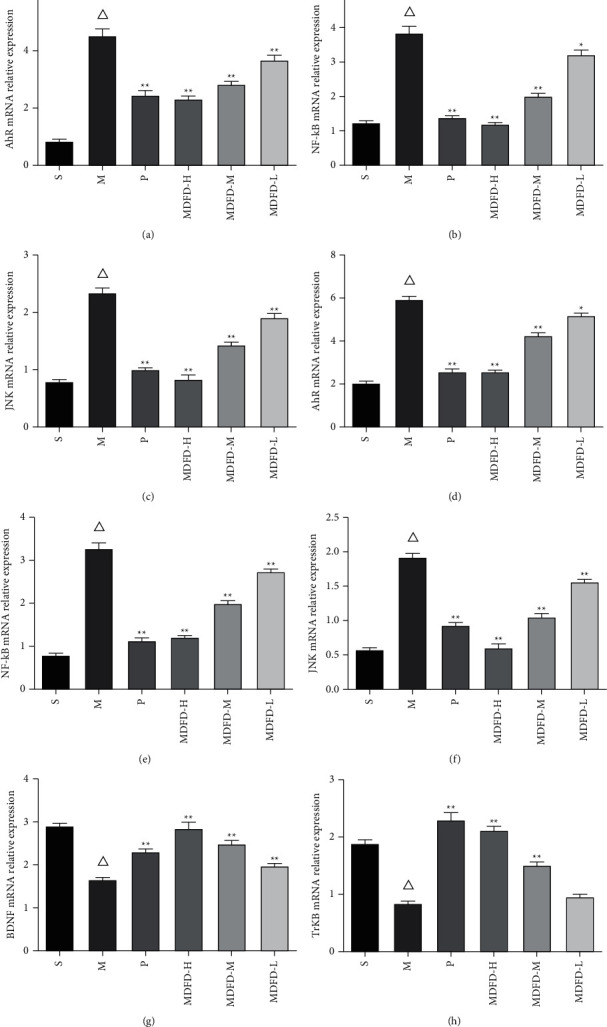
Related mRNA expression levels in the mice brain and kidney of all groups. (a–c) AhR, NF-*κ*B, and JNK mRNA expression levels in the mice kidney. (d–h) AhR, NF-*κ*B, JNK, BDNF, and TrkB mRNA expression levels in the mice brain (Δ*p* < 0.01, comparable to the S group; ^*∗∗*^*p* < 0.01, comparable to the M group; ^*∗*^*p* < 0.05, comparable to the M group; S, sham operation group; M, model group; P, positive control group; MDFD-H, modified Dahuang Fuzi Decoction high-dose group; MDFD-M, modified Dahuang Fuzi Decoction medium-dose group; MDFD-L, modified Dahuang Fuzi Decoction low-dose group; AhR, aryl hydrocarbon receptor; BDNF, brain-derived neurotrophic factor; TrkB, tropomyosin receptor kinase B; NF-*κ*B, nuclear factor-*κ*B; JNK, c-Jun N-terminal kinase).

The results of qRT-PCR also confirmed the above results of immunohistochemistry and immunofluorescence (see Figure 9). Statistically, mRNA expressions of AhR, NF-*κ*B, and JNK in the kidney and brain of mice in group M increased substantially compared to S (*P* < 0.05). In comparison with group M, the mRNA expressions of AhR, NF-*κ*B, and JNK in the kidney and brain of groups (P, MDFD-H, MDFD-M, and MDFD-L) markedly reduced (*P* < 0.05). Moreover, substantial enhanced expression of BDNF and TrkB at mRNA level was observed in the brain of group M mice compared with those in cohort S (*P* < 0.05). Also, BDNF and TrkB expressions (at mRNA level) markedly reduced in the brain of mice in groups (P, MDFD-H, MDFD-M, and MDFD-L) comparable to M (*P* < 0.05) with the intervention effect of different MDFD doses being different.

## 4. Discussion

In recent years, complications of CKD like CI have attracted more and more attention. As a common nephro-vascular toxin, the concentration of IS in serum and brain tissue has been suggested to be closely related to the severity of CI. Iwata [14] confirmed that rats with renal failure showed gradual increase in IS concentration in their brain tissues, which positively correlated with the neurological damage. Yeh [10] enrolled 199 CKD patients with age >50 years old and eGFR <60 mL/min/1.73 m^2^, wherein they detected the serum IS concentration and cognitive function and compared with 84 nonkidney disease patients with the same baseline variables. The results showed that the executive ability score of CKD patients negatively correlated with the serum IS concentration. Lin [15] used the same research method to detect the serum IS concentration of 260 patients with regular hemodialysis and used MMSE and CASI scales to evaluate the cognitive function. The results showed that the serum IS concentration in hemodialysis patients negatively correlated with their cognitive function. It should be pointed out that the above two studies were cross-sectional studies, with the findings being limited in revealing the causal relationship between the parameters. Adesso [16] appropriately cultured IS solution with primary astrocytes of mice, mixed glial cells, and C6 cells. The results showed that combination of IS and AhR site could activate the activities of factors such as proinflammation, NF-*κ*B, and ROS as well as downregulate the levels of neuroprotective mediators (viz., Nrf2 and HO-1), thereby resulting in neuronal apoptosis. Subsequently, the experimenters replaced the IS solution with CKD patients sera, before verifying with IS adsorbent (AST-120). The results showed that the above oxidative reaction and inflammatory pathway mediated by IS were significantly inhibited. Previous researcher [11] added human primary astrocytes to IS for RNA sequencing and expression profile analysis and then confirmed via cell experiments. They observed that IS could increase ROS production, reduce the production of cell protective factors like Nrf2, and induce neuronal apoptosis through reduction of kinase (ERK, MAPK, MEK, p38, and JNK) phosphrylation. Recently, Bobot et al. [17] showed synchronous degeneration of cognitive functions of rats coupled with positive correlation with serum IS level when CKD aggravated. More importantly, IS-induced activation of AhR is an important part of CKD-induced CI. Aforementioned basic experiments mainly focused on animal and cellular model to unearth possible mechanism of CI induced by IS in CKD, but there was little information on drug treatment.

For many years, Chinese herbal medicine has been shown to therapeutically demonstrate unequalled beneficial effect on CKD patients in China [18]. MDFD is a classic prescription that composes of *Radix Aconiti Lateralis Praeparata*, *Radix et Rhizoma Rhei*, and other medicinal plants. In recent years, it has been reported that MDFD treatment can significantly reverse an increase in SCr and BUN levels in patients with CKD. However, there is still unclear therapeutic mechanism of MDFD. In this regard, mice model of CKD with CI complication was established via 5/6 nephrectomized procedure to investigate MDFD effect. Herein, specific inflammatory factors that have been implicated in CKD inflammation were studied. Levels of BUN, KIM-1, SCr, *β*2-MG, SOD, MDA, IL-1 *β*, and TNF-*α* in serum and kidney have usually been associated with CKD renal function [19–21]. The levels of KIM-1, *β*2-MG, MDA, IL-1 *β*, and TNF-*α* in serum and kidney of the model group increased, while SOD activity decreased, amidst serum BUN and SCr levels being increased, thereby indicating that the CRF mouse model was established successfully. After treatment with MDFD, the levels of KIM-1, *β*2-MG, MDA, IL-1*β*, and TNF-*α* in serum and kidney increased, while activity of SOD was enhanced, but serum BUN and SCr levels decreased in a dose-dependent manner. It was thus suggested that MDFD could improve the inflammatory response and enhance the renal function in CKD mice.

Because the brain tissue cells contain more lipids, they are vulnerable to attack by free radicals, which initiate cascade of reactions and consequently culminate in lipid peroxidation damage. SOD is the main antioxidant enzyme in the body, which can protect cells from the damage of active oxides. The level of SOD activity may reflect the ability of the body to remove free radicals and active oxides [22]. Besides the content of lipid peroxidation product, MDA can reflect the extent of damage of tissue cells via free radical attack [23]. IL-1*β* and TNF-*α* and in brain tissue are mainly produced by neurons and glial cells, which are important proinflammatory cytokines with multiple biological effects released earlier after trauma [24]. Other studies have shown that TNF-*α* and IL-1*β* could cause CI [25,26]. We also observed that concentration of MDA, IL-1*β*, and TNF-*α* in the brain of M group raised, while the activity of SOD decreased, in comparison with group S. Thus, these results suggest potential oxidative damage and inflammatory reaction in the brain tissue of CKD mice with CI. High and middle doses of MDFD substantially reduced amounts of MDA, IL-1*β*, and TNF-*α* in the brain of CKD mice with CI but enhanced the activity of SOD. However, the effect of low-dose MDFD was poor. The above results suggest that MDFD could delay the progression of CKD and exhibited remarkable effect on cognitive function improvement in mice with CKD. In this regard, we speculate that MDFD may improve the cognitive function of CKD mice by increasing the concentration of IS, thereby inhibiting the production of lipid peroxides, improving the scavenging effect of free radicals, and regulating the inflammatory response as reported by Adesso [16]. We employed the MWM test to evaluate cognitive function of the mice because of its sensitiveness to testing of memory and spatial learning abilities. The results showed that high and middle doses of MDFD had significant effect on improving CI in CKD mice, which was close to prednisone.

To find out the mechanism of MDFD on cognitive function of CKD mice, we further detected the expression of NF-*κ*B, AhR, and JNK in kidney and brain and TrkB and BDNF in brain only. AhR is a powerful ligand of IS. In the brain, AhR can mainly be found in the hippocampus, cerebellum, and cerebral cortex [27] and is associated with sensory and CI caused by excitotoxicity and oxidative stress [28, 29]. Other studies have shown that IS could promote oxidative reaction and induce apoptosis of nerve cells after activating AhR in nerve cells [6]. A transcription factor in the nucleus, NF-*κ*B is shown to regulate proinflammatory cytokine genes [30]. Feng [31] found that neuronal apoptosis mediated by NF-*κ*B is an underlying factor, which caused cognitive dysfunction, therein becoming an important target for medications of the disease. The ability IS-induced ROS to influence NF-*κ*B activation has been discussed elsewhere [32]. Regulation of cellular survival and death by MAPK pathway was found by bioinformatics analysis to be one of the principal pathways in cell signaling [33]. MAPK has three major families, namely, ERK, JNK, and p38MAPK. Stress response is mediated by JNK, which is considered a crucial signal pathway and mediate neuronal apoptosis and regeneration [34]. It was generally believed that JNK signaling pathway is closely related to apoptosis [35]. More evidences have shown that NF-*κ*B had complex interactions with JNK pathway and is responsible for the regulation of apoptosis in several cell types [36, 37]. Our results showed that AhR, NF-*κ*B, and JNK proteins increased substantially in the kidney and brain of the model group. Fortunately, after MDFD treatment, AhR, NF-*κ*B, and JNK proteins markedly decreased in the kidney and brain of CKD mice. This suggests that inhibition of AhR/NF-*κ*B/JNK signaling pathway might serve as mechanistic action of MDFD through which it improved CI in CKD mice. Moreover, data of qRT-PCR also reflected the same phenomenon.

As a significant neurotrophic family member, BDNF is a protein with 119 basic amino acids [38]. The biological activity of BDNF mainly depends on its receptor TrkB. After BDNF specifically binds to TrkB receptor on cell membrane, it can promote neuronal proliferation and apoptosis by activating downstream signal molecules and participating in the regulation of cognitive function [39]. BDNF regulates transmission and plasticity of synapses in multiple regions of central nervous system in the brain during adulthood, thus participating in the formation of learning and memory, improving memory, and restoring impaired cognitive function [40]. It was found that the expression of TrkB protein in BDNF knockout mice significantly decreased, while the learning ability of mice was impaired [41]. Corroboratively, results of immunohistochemistry, immunofluorescence, WB, and qRT-PCR analyses showed a decrease in levels of related proteins of cognitive function in CKD mice brain, but their expression was upregulated after MDFD treatment. The above results could suggest that MDFD protected neurons by inhibiting IS-mediated AhR/NF-*κ*B/JNK signaling pathway, which may be achieved by enhancing the activity of BDNF/TrkB signaling pathway.

## 5. Conclusions

We basically identified 61 main chemical components in MDFD by LC-MS. MDFD reduced serum levels of oxidative and inflammatory markers but increased endogenous antioxidants in treated mice. In addition, MDFD improved the cognitive function of CKD mice with CI. It also ameliorated the injured kidney and brain. These results support the possibility of MDFD playing a protective role by supplementing the therapeutics of CKD-CI. This study also suggests that MDFD improved CKD-CI, which might be attributed to the upregulation of AhR/NF-*κ*B/JNK pathways. Therefore, our finding provided new insights into mechanistic action of MDFD on CKD mice with CI. Nevertheless, further works should explore the prospect of developing MDFD as a candidate drug for CKD patients with CI.

## Data Availability

The datasets generated during and/or analysed during the current study are available from the corresponding author on reasonable request.

## Supplementary Materials

Supplementary Materials Figure S1: immunohistochemistry measurements of expression levels of related proteins in the mice brain and kidney of all groups. Figure S2: immunofluorescence measurements of expression levels of related proteins in the mice brain and kidney of all groups.
