# Supplementary material for: Treatment of Modified Dahuang Fuzi Decoction on Cognitive Impairment Induced by Chronic Kidney Disease through Regulating AhR/NF-κB/JNK Signal Pathway
Source: Evid Based Complement Alternat Med. 2022 Apr 14;2022:8489699. doi: 10.1155/2022/8489699 (PMC9023153; doi:10.1155/2022/8489699)
Supplement: Supplementary Materials — Figure S1: immunohistochemistry measurements of expression levels of related proteins in the mice brain and kidney of all groups. Figure S2: immunofluorescence measurements of expression levels of related proteins in the mice brain and kidney of all groups. [file 8489699.f1.docx]

**Treatment of Modified Dahuang Fuzi decoction on cognitive impairment induced by chronic kidney disease through regulating AhR/NF-κB/JNK signal pathway**

Mingjia Gu *^a,^*^#^, Pu Ying *^a,#^*, Zhiwei Miao *^c,^*^#^, Xiang Yu *^b^*, Rui Bao *^d^*, Jian Xiao *^a^*, Leiping Gao *^a,*^* , Juping Chen *^a,*^*

*^a^* *Changshu Hospital Affiliated to Nanjing University of Chinese Medicine, Changshu, Jiangsu province 215500, P.R. China.*

*^b^ Nanjing University of Chinese Medicine, Nanjing, Jiangsu province 210046, P.R. China.*

*^c^* *Zhangjiagang TCM Hospital Affiliated to Nanjing University of Chinese Medicine, Zhangjiagang, Jiangsu province 215600, P.R. China.*

*^d^ The College of Pharmacy of Jiangsu University, Zhenjiang, Jiangsu province 212000, P.R. China.*

^#^ These authors contributed equally to this work.

^*^Co-corresponding Author: Juping Chen & Leiping Gao (E-mail: Gaolp2020@163.com)

Address: Changshu Hospital Affiliated to Nanjing University of Chinese Medicine, Changshu, Jiangsu province 215500, P.R. China.

## Methods

## Immunohistochemistry

Levels of AhR, NF-κB, JNK, BDNF and TrkB in brain, and AhR, NF-κB and JNK protein expression in kidney were detected by immunohistochemistry. The hippocampus and kidney of each group were sectioned (4 μm). For tight adherence of the slides prior to dewaxing to remove all the moisture content, the slices were heated for 30-60 min in an oven at 60℃. Soaking (twice) of the slides in xylene was done for 10 min (each time). Then, the slices were soaked again in gradient ethanol (100%, 95%, 90%, 80%, 70%, 50% and 30%), water, PBS and 3% H2O2 by turn for 5 min, respectively. Washing (thrice) of the slices was carried out with PBS for 5 min (each time) before they were soaked (twice) in boiling repair buffer for 5 min (each time). Next, similar procedure was followed to wash the slides with PBS before they were put into 5% BSA sealing solution for 20 min. Then, the samples were incubated overnight at 4℃ after addition of 50 μL primary antibody and rewarming at 37℃ for 30 min. Addition of secondary antibody (50 µL) and incubation (for 30 min) at 37℃ were carried out after 5 min (each time) and then PBS washing (twice) of the slides was carried out. Afterwards, similar procedure for washing the slides was followed before addition and incubation of SABC for 30 min at 37℃. Next, the sides were washed as stated above and DAB solution was used to develop the color for 15~30 min, while excess dye liquor was washed away with distilled water. Finally, it was re-dyed with hematoxylin, dehydrated and sealed, before it was finally observed under microscope (Nikon, Japan).

## Immunofluorescence

Detection of the expression of protein expression levels of AhR, NF-κB, JNK, BDNF, TrkB proteins in brain, as well as NF-κB, AhR and JNK proteins in kidney was carried out via immunofluorescence assay. Next, 4 μm paraffin section was made from hippocampus and kidney of mice in each group. The slices were dewaxed with xylene and washed with gradient ethanol. The antigen was extracted by citric acid buffer microwave method. After washing and drying with PBS, 5% BSA was sealed for 30 min. After removing BSA solution of the slides, 0.5% Triton-x100 was added prior to incubation (30 min), and subsequent PBS washing (thrice) for 5 min (each time). Each section was covered with 50 μL diluted primary antibody and kept moist overnight at 4 ℃. After rewarming at 37 ℃ for 2 h, each slice was washed based on above-described procedure. Next, 50-100 μL of the corresponding secondary antibody was added to each slice, and kept for 2~3 h at 37 ℃. Each slice was washed with PBS prior to the addition of 50-100 μL DAPI dye solution and subsequent incubation in the dark for 15 min (ambient temperature). After staining, washing of the sections was carried out with PBS as stated above and the appropriate amount of anti-fluorescence quenching agent was added to the tissues. The slides were covered, and the fluorescence microscope (Nikon, Japan) was used to observe and take photos.

## Results

The AhR, NF-κB and JNK proteins were observed to be consistently expressed in mouse kidney (Figure S1 A-C). In group S, only a few positive neurons seen in the hippocampal area were all stained with light cytoplasm and displayed single shape. Observation of more positive cells in hippocampus of mice in group M, showed that most of those cells were stained in the nucleus, amidst large morphological variation. Most of the positive cells were circular in shape and mainly neurons. The expression of positive cells in hippocampus of P and MDFD high-dose treatment groups was consistent with that of group S, while the expression of middle dose MDFD treatment group was comparable to groups S and M. However, insignificant difference was observed between groups (low-dose MDFD and M).

The expression of AhR, NF-κB, JNK, BDNF and TrkB proteins in brain were revealed in Figure S1 D-H. Compared with group S, the positive cells of AhR, NF-κB and JNK in M increased substantially, but those in BDNF and TrkB decreased markedly compared to S. The number of AhR, NF-κB and JNK immunoreactive cells in the groups, (namely P, MDFD high and medium dose) significantly decreased, while BDNF and TrkB immunoreactive cells increased markedly in comparison with group S.

Also, AhR, NF-κB and JNK expression (at proteins level) in kidney of mice are shown in Figure S2 A-C. Nucleus (DAPI) localization was indicated by blue fluorescence while AhR, NF-κB and JNK localizations were shown by green fluorescence. Compared with group S, a large number of green fluorescence was observed in renal cells of mice in group M under fluorescence microscope, which indicated that the AhR, NF-κB, JNK protein expression in renal cells of CKD mice was increased. After the intervention of prednisone and high and middle doses of MDFD, expression of AhR, NF-κB and JNK proteins in renal of CKD mice decreased, amidst insignificant difference between MDFD-L and M groups. As shown in Figure S2 D-H, compared with group S, expression of AhR, NF-κB, and JNK expression in the brain of group M increased. Also, AhR, NF-κB and JNK at protein level in brain of the groups (P, MDFD-H and MDFD-M) reduced in comparison with group M, but the comparison between MDFD-L and M groups showed statistically insignificant difference. In contrast, the BDNF and TrkB expression (at protein level) in group M decreased compared with group S, but it increased in groups (P, MDFD-H and MDFD-M) compared to group M.

## Figures


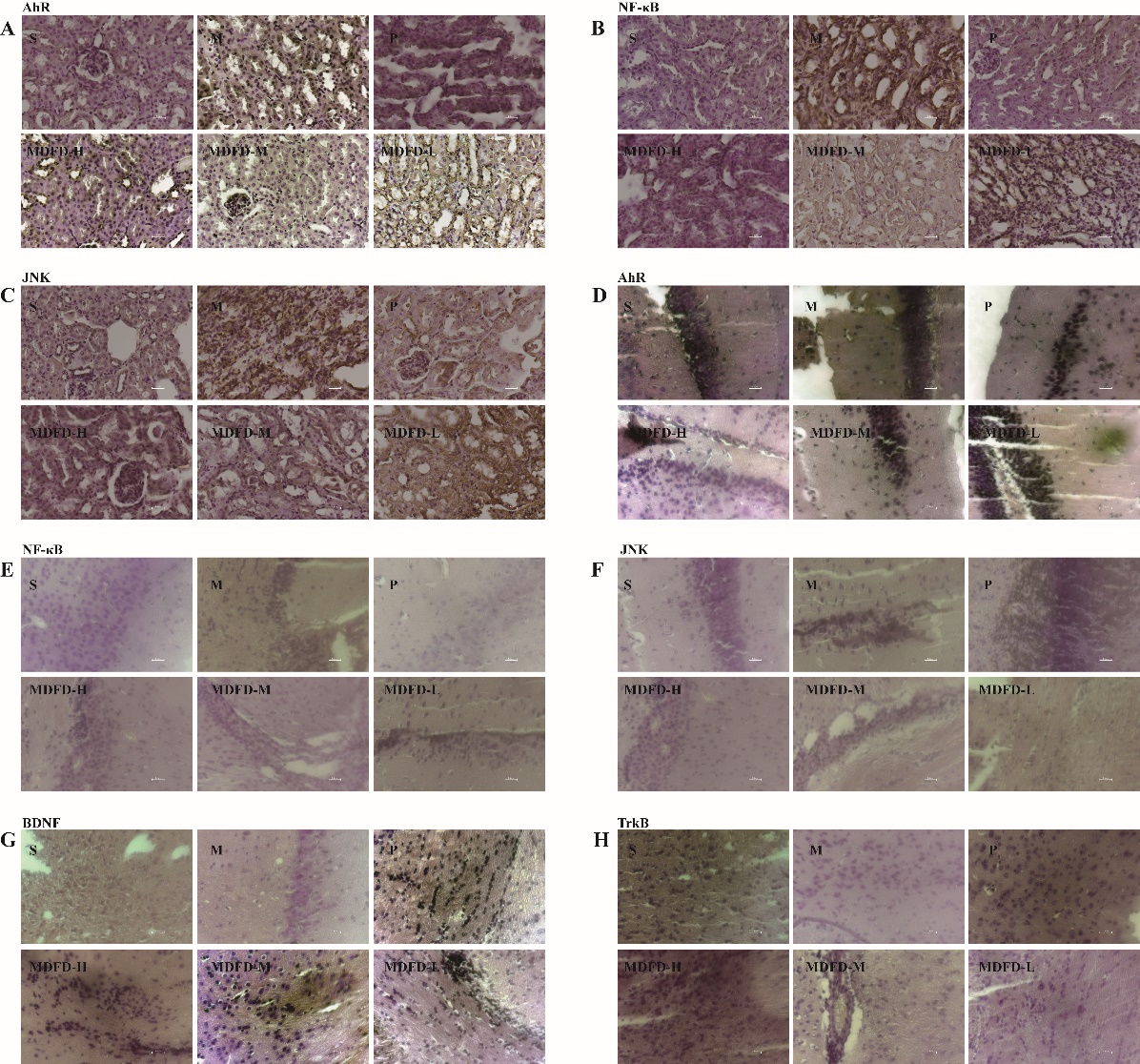
Figure S1. Immunohistochemistry measurements of expression levels of related proteins in mice brain and kidney of all groups. (A-C) AhR, NF-κB and JNK protein expression levels in mice kidney; (D-F) AhR, NF-κB and JNK protein expression levels in mice brain; (G-H) BDNF and TrkB protein expression levels in mice brain. (S, sham operation group; M, model group; P, positive control group; MDFD-H, Modified Dahuang Fuzi Decoction high dose group; MDFD-M, Modified Dahuang Fuzi Decoction medium dose group; MDFD-L, Modified Dahuang Fuzi Decoction low dose group; AhR, aryl hydrocarbon receptor; BDNF, brain-derived neurotrophic factor; TrkB, tropomyosin receptor kinase B; NF-κB, nuclear Factor-κB; JNK, c-Jun N-terminal kinase.)


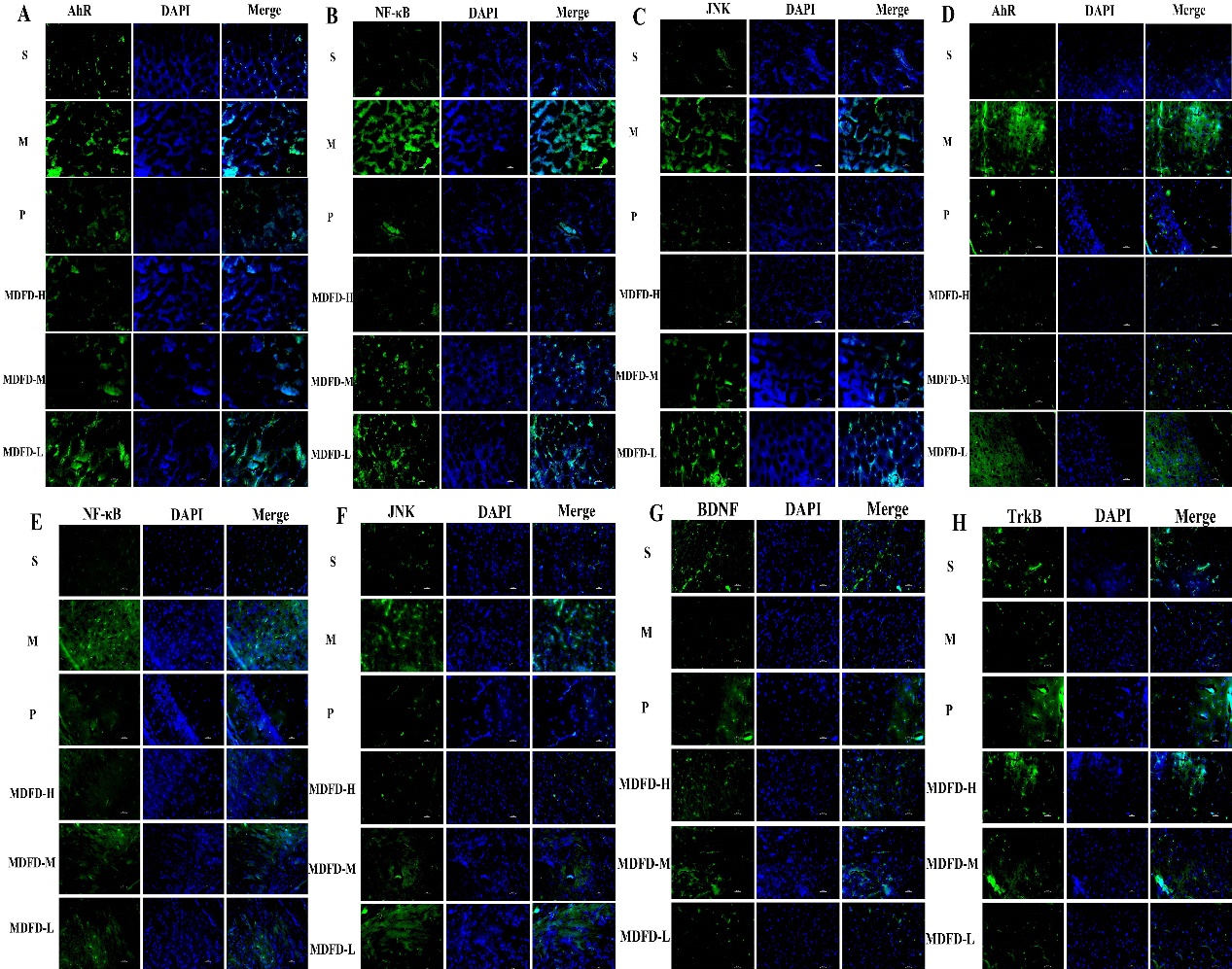
Figure S2. Immunofluorescence measurements of expression levels of related proteins in mice brain and kidney of all groups. (A-C) AhR, NF-κB and JNK protein expression levels in mice kidney; (D-F) AhR, NF-κB and JNK protein expression levels in mice brain; (G-H) BDNF and TrkB protein expression levels in mice brain. (S, sham operation group; M, model group; P, positive control group; MDFD-H, Modified Dahuang Fuzi Decoction high dose group; MDFD-M, Modified Dahuang Fuzi Decoction medium dose group; MDFD-L, Modified Dahuang Fuzi Decoction low dose group; AhR, aryl hydrocarbon receptor; BDNF, brain-derived neurotrophic factor; TrkB, tropomyosin receptor kinase B; NF-κB, nuclear Factor-κB; JNK, c-Jun N-terminal kinase.)
